# Looking for the Optimal PD-1/PD-L1 Inhibitor in Cancer Treatment: A Comparison in Basic Structure, Function, and Clinical Practice

**DOI:** 10.3389/fimmu.2020.01088

**Published:** 2020-05-29

**Authors:** Yu Chen, Yanqing Pei, Jingyu Luo, Zhaoqin Huang, Jinming Yu, Xiangjiao Meng

**Affiliations:** ^1^Cheeloo College of Medicine, Shandong University, Jinan, China; ^2^Department of Radiation Oncology, Shandong Cancer Hospital and Institute, Shandong First Medical University and Shandong Academy of Medical Sciences, Jinan, China; ^3^Department of Radiology, Shandong Provincial Hospital Affiliated to Shandong First Medical University, Jinan, China

**Keywords:** PD-1 inhibitors, PD-L1 inhibitors, comparison, differences, optimal treatment, efficacy

## Abstract

Programmed cell death protein-1/ligand 1 (PD-1/L1) targeted immune checkpoint inhibitors have become the focus of tumor treatment due to their promising efficacy. Currently, several PD-1/PD-L1 inhibitors have been approved for clinical practice with several more in clinical trials. Notably, based on available trial data, the selection of different PD-1/PD-L1 inhibitors in the therapeutic application and the corresponding efficacy varies. Widespread attention then is increasingly raised to the clinical comparability of different PD-1/PD-L1 inhibitors. The comparison of the inhibitors could not only help clinicians make in-depth understanding of them, but also further facilitate the selection of the optimal inhibitor for patients in treatment as well as for future clinical research and the development of new related drugs. As we all know, molecular structure could determine molecular function, which further affects their application. Therefore, in this review, we aim to comprehensively compare the structural basis, molecular biological functions, and clinical practice of different PD-1/PD-L1 inhibitors.

## Background

PD-1/PD-L1 inhibitors have created a paradigm shift in cancer immunotherapy. Binding of PD-1 to its ligand PD-L1 can trigger an inhibitory signal, leading to reduced T-cell proliferation, and anti-tumor immunity. Blocking the binding of PD-1 to PD-L1, has been shown to reinvigorate T-cell activity and the anti-tumor immune response, which supports the rationale for PD-1/PD-L1 inhibitors as promising therapeutics. Until now, PD-1 inhibitors (nivolumab and pembrolizumab) and PD-L1 inhibitors (atezolizumab, avelumab, and durvalumab) have been approved by the US Food and Drug Administration (FDA) for the treatment of a wide spectrum of tumors, including NSCLC, urothelial cancer, melanoma, head and neck squamous cell cancer, and lymphoma (1). In addition, a number of newly engineered PD-1/PD-L1 inhibitors are undergoing clinical trials in the hope of achieving improved clinical outcomes (2–4).

Because a variety of PD-1/PD-L1 inhibitors are available for cancer treatment, it is of great clinical significance to have a comprehensive understanding of the potential differences between these agents, to enable the selection and development of optimal treatments. Here, we illustrate the rationale of action mechanism, describe the structural basis of the PD-1 and PD-L1 inhibitors, IgG4 and IgG1, respectively, make a comparison in terms of their molecular structure, biological function, and clinical perspectives and further highlight future research needs to achieve optimal patient management and the development of new related drugs.

## Rationale OF PD-1/PD-L1 Inhibitors

**Figure 1:**
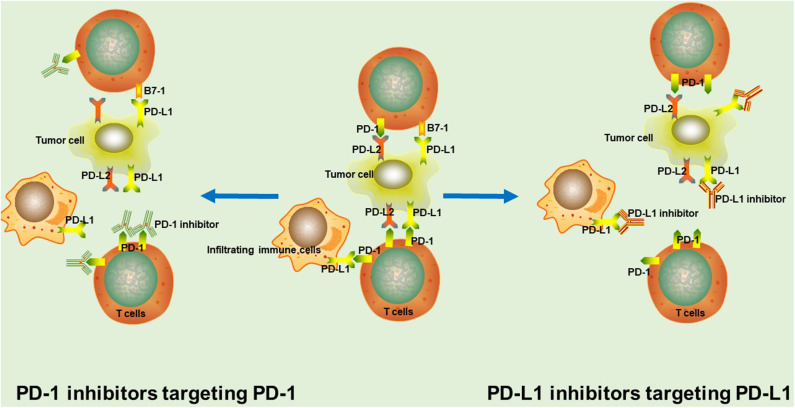
The rationale of PD-1/PD-L1 inhibitors. In tumor tissue, PD-1 interacts with PD-L1 or PD-L2 to mediate significant immune suppression. PD-1/PD-L1 inhibitors binds to corresponding target, thus blocking the PD-1/PD-L1 signaling pathway and markedly enhancing T cell function and the anti-tumor immunity.

Tumor-specific cytotoxic T cells are capable of recognizing cancer neoantigens, further inducing tumor cell death through direct killing, and the release of inflammatory mediators. During this process, it has been found that activated T cells upregulate the expression of PD-1 on their surface. PD-1 is a monomeric type I immune inhibitory transmembrane receptor, mainly expressed in T cells, B cells, natural killer cells, and many other tumor-infiltrating lymphocytes (5). Likewise, the PD-1 ligand, PD-L1, is a type I transmembrane protein, normally expressed on antigen-presenting cells as well as non-immune cells, such as Kupffer cells and epithelial cells (6, 7). Generally, the interaction between PD-1 and PD-L1 not only prevents excessive lymphocyte activation and achieves immune tolerance to self-antigens, but it also downregulates the anti-tumor function of T cells, thus leading to tumor immune escape (5, 8). Therefore, it has been observed that most tumors upregulate PD-L1 expression in response to interferon-γ (IFN-γ) and other inflammatory mediators, thus delivering enhanced inhibitory signals to T cells and resulting in poor survival rates (9). Overactivation of the PD-1/PD-L1 pathway in tumors and the resulting poor patient survival rates, makes this pathway a feasible target for antibody treatment (Figure 1).

PD-1/PD-L1 inhibitors are used to shift the balance toward immune activation, further enhancing tumor immunosurveillance, and anti-tumor immune responses. PD-1 inhibitors interact with PD-1 through overlapping surface regions, which prevents the binding of PD-L1 or PD-L2 to PD-1 (10). Nivolumab and pembrolizumab are the two most representative PD-1 inhibitors that have already been approved by the US FDA for the clinical treatment of a variety of tumors (11). Besides, many other PD-1 inhibitors, such as sintilimab, tislelizumab and camrelizumab (SHR-1210) are currently being studied in clinical trials and have achieved significant survival benefits (2, 3, 12). Anti-PD-L1 monoclonal antibodies inhibit the binding of PD-L1 with PD-1 and B7-1 on T cells (13, 14). Up to now, atezolizumab, durvalumab, and avelumab are the most promising PD-L1-targeting drugs for cancer treatment (11).

## IGG, the Molecular Structure Basis OF PD-1/PD-L1 Inhibitors

**Figure 2:**
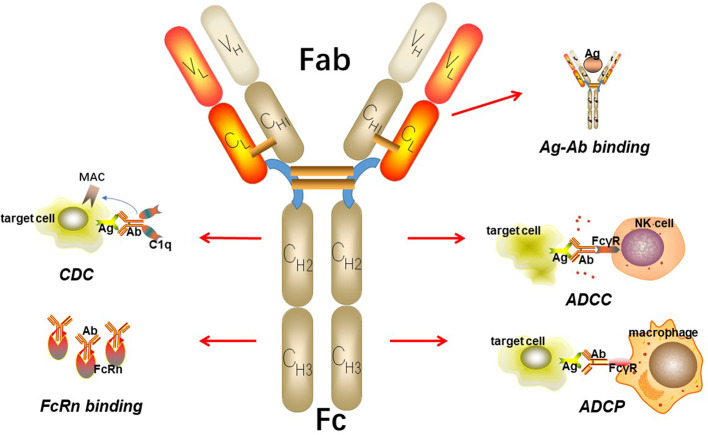
The basic structure of IgG. IgG can be divided into two parts including Fab fragment and Fc fragment. Fab fragment plays an important role in antigen recognition and binding while Fc fragment can mediate ADCC, CDC, and ADCP. Besides, Fc fragment can bind to FcRn to protect itself from elimination.

IgGs are made up of two heavy chains and two light chains, which connect to each other by inter-chain disulfide bridges (Figure 2). Based on enzymatic digestion with papain, an antibody can also be divided into two Fab regions, which play an indispensable role in antigen recognition and one Fc region that mediates antibody effector functions, such as antibody-dependent cellular phagocytosis (ADCP), antibody-dependent cell-mediated cytotoxicity (ADCC), and complement-dependent cytotoxicity (CDC) by binding to its receptor, FcγR (15–17). The target-specific binding and immune-mediated effector functions shed light on the promising role of IgG as the basic framework for engineering recombinant monoclonal antibodies.

According to variations in the length and sequence of the hinge region, and sequence variation in the Cγ2 and Cγ3 domains of heavy chains, human IgG antibodies are further divided into four different subtypes including IgG1, IgG2, IgG3, and IgG4, among which IgG4 is the least while IgG1 is the most (18, 19). These four subtypes share a similar structure, with ≥90% sequence homology (20). Nonetheless, single amino acid differences can give rise to unique profiles of structure and effector function among subtypes (Table 1).

**Table 1:** Comparison of the four human IgG subtypes.

	**IgG1**	**IgG2**	**IgG3**	**IgG4**
Percentage of the total IgG	60	25	10	5
Amino acid in hinge region ^(19)^	15	12	62	12
Inter-heavy chain disulfide bonds	2	4	11	2
Antibody-dependent cell-mediated cytotoxicity (ADCC) ^(17)^	+++	+/–	++	+/–
Complement-dependent cytotoxicity (CDC)	++	–	+++	–
Antibody-dependent cell-mediated phagocytosis (ADCP)	+	+	+	+/–
FcRn binding ^(21)^	+	+	+	+
Stability ^(22)^	Stable	Covalent dimer	Prone to protease digestion	Fab arm exchange
Half-life (d)	21–23	20–23	7–8	21–23

IgG1 is the most abundant of the four human IgG subclasses, making up ≥50% of the total serum IgG concentration. IgG1, with a stable structure, could induce potent effector functions mediated by the Fc fragment, thus achieving cytotoxic or apoptotic responses. PD-L1 inhibitors recognize and bind to PD-L1 on tumor cells, further blocking the PD-1/PD-L1 signaling pathway. To enhance the anti-tumor response, PD-L1 inhibitors must bind to FcγR and C1q with high affinity to evoke potent ADCC and CDC to clear tumor cells, which indicates that IgG1 is the optimal structural basis of PD-L1 inhibitors. Nonetheless, in clinical development, durvalumab and atezolizumab are engineered to elicit a weakened effector function, to protect PD-1/PD-L1 double-positive immune cells and reduce potential adverse effects (23, 24). However, avelumab still maintains the capability to activate effective ADCC (25).

IgG4 is the least common IgG subclass, accounting for ~5% of the total serum IgG concentration (18). Due to its low affinity for C1q and FcγR, IgG4 only weakly induces CDC and ADCC. Typically, PD-1 inhibitors recognize and bind to PD-1 on activated T cells, thus shifting the balance to immune activation. To avoid Fc-mediated cytotoxic effects on T cells, PD-1 inhibitors are supposed to have attenuated effector functions, with low affinity for FcγR and C1q. IgG4 with a lower propensity to elicit effector functions is designed to block ligand-receptor interactions instead of killing antigen-expressing target cells, which makes it the optimal IgG subtype to target PD-1 on T cells for a favorable anti-tumor response (26). Of particular concern, IgG4 behaves as a monovalent-bispecific antibody as a result of the *in vivo* exchange of IgG half-molecules, a process termed Fab-arm exchange (FAE) (22, 27). It has been demonstrated that the change of a proline (Pro) in the core hinge to a serine (Ser) leads to the formation of “intra-chain” disulfide bonds instead of “inter-chain” disulfide bonds, as well as attenuated non-covalent interactions between the heavy chains in the CH3 region, thus resulting in the dissociation and recombination of heavy chains and further generating newly bi-specific heterozygous IgG4 with reduced avidity to antigen (28–30). Therefore, the core hinge Ser228Pro (S228P) mutation is a significant design consideration for therapeutic IgG4 antibodies to abrogate FAE (31, 32). Pembrolizumab, containing a S228P mutation, is a compact molecule with an asymmetrical Y shape and a short hinge region. The Fc domain is glycosylated at Asn297 in the CH2 domain on both chains with one CH2 domain rotated 120°, causing the corresponding glycans to face the solvent (32). Another PD-1 inhibitor, nivolumab, also with an S228P mutation, has a structure nearly identical to pembrolizumab, except for the variable regions, which serve the functions of antigen recognition and binding.

## Comparison in Biological Function Among PD-1/PD-L1 Inhibitors

### Structural Mechanism of Action

Based on X-ray crystal structure analysis, the general interaction between PD-1 and PD-L1 is mediated mostly by the residues of the C′CFG strands within both molecules. This binding covers a buried surface area of 1,970 Å^2^ and triggers a moderate conformational change in the PD-1 CC′ loop, which makes it capable of closing around PD-L1 (21). The dissociation constant, K_D_, is usually used to reflect the affinity of molecular interactions. Typically, a higher K_D_ value indicates a lower affinity. The K_D_ value for PD-1 binding to PD-L1 is ~8.2 μM.

PD-1 inhibitors can competitively bind to PD-1 with PD-L1 because of sharing an overlapping binding surface. Of note, although they have a similar molecular mechanism of action, PD-1 inhibitors have significant differences with respect to how they interact with PD-1 (Table 2). Nivolumab binds to PD-1 using the residues of the N-terminal extension, accompanied by contributions from both the FG and BC loops of the IgV domain. This binding covers a buried surface area of 1,487–1,932.5 Å^2^, with an overlapping binding area for nivolumab and PD-L1, which is mainly located on the FG loop. Notably, the N-loop, which is not involved in PD-L1 recognition, is mainly responsible for binding affinity with a correlated K_D_ value about 3.06 nM (34). As for pembrolizumab, the interaction with PD-1 mainly depends on the flexible C′D loop of PD-1, but the C, C′, and F strands are involved as well. The total buried binding surface area is 1,774–2,126 Å^2^, with the competing binding area of pembrolizumab and PD-L1 mainly located on the FG loop. The K_D_ value for this interaction is ~29 pM, which is mainly influenced by the C′D loop (10, 35). Apart from direct occupancy, it has been demonstrated that, after binding to PD-1, both inhibitors can trigger slight conformational changes in the flexible BC and FG loops, thus further potently inhibiting PD-L1 binding (21). Of particular interest, given the fact that there is nearly no overlapping antigen binding site on PD-1 for these two inhibitors, it has been speculated that the simultaneous administration of pembrolizumab and nivolumab may have the potential for superior therapeutic efficacy. However, more research is needed to test this hypothesis.

**Table 2:** Comparison of the different PD-1/PD-L1 inhibitors.

**Drug**	**Nivolumab**	**Pembrolizumab**	**Atezolizumab**	**Avelumab**	**Durvalumab**
**Subtypes**	**IgG4**	**IgG4**	**IgG1**	**IgG1**	**IgG1**
Binding area	N-loop	C'D loop	CC′FG strands	F and G strands	the N-terminal region, the CC' loop and the CC′FG strands
Buried surface ^(33)^	1,487–1,932.5 Å	1,774–2,126 Å	2,106 Å	1,815 Å	1,624 Å
Affinity	3.06 nM	29 pM	1.75 nM	0.046 nM	0.66 nM
Degree of humanization	Fully human	Humanized	Fully human	Fully human	Fully human
Immunogenicity	12.9%	1.8%	NR	NR	0.4%
Modification	S228P	S228P	Fc engineering	-	Fc engineering
Distribution	Spleen	Lung, liver, kidney, spleen	NR	NR	NR
Half life ^(33)^	26.7	25.8	27	6	17
Dosage regimen	3 mg/kg q2w	200 mg q3w	1,200 mg q3w	10 mg/kg q2w	10 mg/kg q2w

The main interaction between PD-1 inhibitors and PD-1 is dependent on the flexible loops of PD-1, which are typically not part of the PD-1/PD-L1 binding surface. By contrast, PD-L1 inhibitors can bind to PD-L1 using the epitope that significantly overlaps with the PD-1 binding interface (36). Avelumab binds to PD-L1 via the epitope located mainly on the F and G strands of PD-L1, partially overlapping with the PD-1 binding region, with a K_D_ value of ~42 pM (37). For atezolizumab, the CDRs located in the VH chains dominate the binding, mainly by covering the CC′FG strands and residues in the BC, CC′, C′C′′, and FG loops (38). The associated K_D_ value is ~400 pM (39). In addition, the binding sites of durvalumab are mainly located in the N-terminal region, the CC′ loop and the CC′FG strands, with a K_D_ value of about 667 pM (40). Notably, in contrast to PD-1 inhibitors, PD-L1 inhibitors do not usually induce a significant conformational change of PD-L1.

### Immunogenicity

As protein therapeutics, antibodies are inevitably immunogenic, which induces the production of anti-drug antibodies (ADA), thus affecting the safety and efficacy by antibody-mediated neutralization or hypersensitivity responses (41, 42). Hence, immunogenicity evaluation is required for all antibodies during product engineering. Of note, the immunogenicity of antibodies is regulated by a variety of factors, including structural characteristics, manufacturing techniques, drug-delivery methods, and patient immune status, among which, structural characteristics are the most important.

To overcome the high immunogenicity of complete murine-origin antibodies, the preparation of monoclonal antibodies has gone through several evolutionary processes. Chimerization was first attempted by combining mouse antigen-binding domains with human constant region domains, to form chimeric mouse-human antibody molecules, with ~67% of the primary sequence of the antibody derived from the human sequence (43). Humanization was the following development, in which the CDRs from the heavy-chain variable region of mouse IgG were grafted onto the human IgG structure, to create a humanized monoclonal antibody with ~95% human-derived sequence (44). Recent technological breakthroughs have enabled the generation of fully human antibodies from transgenic mice that have been genetically engineered with the human IgG locus (45). Nivolumab, together with atezolizumab, sintilimab, avelumab, durvalumab, and tislelizumab are fully human PD-1/PD-L1-targeting monoclonal antibodies, while pembrolizumab, JS-001, and camrelizumab are humanized antibodies. It remains unclear whether fully human antibodies show lower immunogenicity compared to humanized antibodies. One certainty is that fully human antibodies do show at least some immunogenicity, possibly attributed to unique idiotypes, unique post-translational modifications, or impurities related to the manufacturing process (46).

The detection of ADAs can reflect the immunogenicity of agents to some extent. According to the instructions, in patients treated with nivolumab, 12.9% (287 of 2,232) tested positive for ADAs, while the rate of neutralizing antibody detection was ~0.7% (16 of 2,232). In patients treated with pembrolizumab, the corresponding rate of ADAs was 1.8% (36 of 2,034) with neutralizing antibodies detected at a rate of 0.4% (9 of 2,034). Moreover, in a phase 1/2 trial of stage IIIB–IV NSCLC, 3.1% (26 of 849) of patients treated with durvalumab tested positive for ADAs, while neutralizing antibodies were detected in only 0.4% of patients (3 of 849) (47). Thus, far, due to limited research, it is unclear whether treatment-emergent ADAs have any impact on the pharmacokinetics (PK), efficacy, or safety of related drugs. When comparing the rate of ADA positivity for different PD-1/PD-L1 inhibitors, in addition to the intrinsic effects of the drugs, the sensitivity and specificity of the ADA detection methods, sample handling and collection, the combination of other drugs and the underlying disease status of the patients should all be taken into consideration.

### Pharmacokinetics and Dosage Regimen

Pharmacokinetic research is indispensable before a drug can be applied to clinical practice. Pharmacokinetics (PK) usually involves four fundamental aspects: absorption, distribution, metabolism, and elimination. As macromolecule-protein biologics, PD-1/PD-L1 inhibitors are administered intravenously that is considered 100% absorption. Typically, the distribution of the drugs is limited because large molecules do not spread efficiently from the blood to peripheral compartments (48). The specific metabolic mechanisms involved include hepatic metabolism and direct proteolytic degradation, with the latter playing a major role (48). *In vivo* non-human primate imaging using a ^89^Zr-nivolumab tracer, in a study performed by Cole et al., showed that the spleen is the main organ of distribution, with primary clearance through the liver (49). While for pembrolizumab, the volume of distribution at a steady state is 7.4 L (coefficient of variation: 19%), with a consistently restricted extravascular distribution mainly concentrated in the lung, liver, kidney, and spleen.

Notably, elimination appears to play a dominant role in PK and is under the influence of various regulation such as neonatal Fc receptor (FcRn)-dependent recycling, target-mediated drug disposition (TMDD), and non-specific or off-target binding-mediated clearance, among which FcRn-dependent recycling plays a major role (50, 51). It has been demonstrated that TMDD leads to rapid clearance by inducing internalization and downstream degradation (50). On the contrary, FcRn binding to IgG via its Fc CH2-CH3 interface can rescue it from elimination, resulting in an extended half-life (52, 53). Due to restricted FcRn expression, FcRn-dependent recycling is capacity limited. It has been observed that high concentrations of IgG can saturate the FcRn recycling system, thus decreasing recycling efficiency and resulting in a significant increase in the fractional catabolic rate of IgG. This should be taken into consideration when choosing the optimal dosage regimen (50).

The antibodies currently on the market are a little different in their *in vivo* PK. Generally, the standard dosage regimen for clinical practice needs to be based on a comprehensive understanding of the PK. Due to different PK, the dosage regimens for PD-1 and PD-L1 blockade vary. A nivolumab dosage regimen of 3 mg/kg every 2 weeks exhibited superior efficacy while 200 mg of pembrolizumab every 3 weeks has already been defined as the standard dosage regimen for the treatment of various tumors (54–57). For atezolizumab, based on various research data, the currently recommended dosage regimen for further clinical development is 1,200 mg (15 mg/kg) (58). To achieve a target occupancy of 90%, a dosage regimen of 10 mg/kg every 2 weeks is optimal for avelumab treatment (59). Moreover, durvalumab can achieve maximum efficacy when applied with 10 mg/kg every 2 weeks (60).

A relatively long half-life allows drugs to work in the body for a longer period, thus allowing relatively less frequent dosing. It may be possible to reduce dosing frequencies by engineering the PK of drugs to give them a relatively long half-life, which would finally make them convenient and cost-effective.

## Clinical Comparison of PD-1/PD-L1 Inhibitors

### Predictive Biomarkers

Although PD-1/PD-L1 inhibitors have made a great success in the management of a broad range of cancer types, most patients are intrinsically resistant and fail to respond well. In the era of precision treatment, it's of great significance to identify patients who are more likely to benefit from immunotherapy.

PD-L1 and tumor-mutation burden (TMB) are currently the two most promising predictive biomarkers. Generally, the overexpression of PD-L1 or a high TMB may correlate with better objective response, durable clinical benefit, and longer progression-free survival (PFS) (33, 61). However, it has been found that the predictive role of the two for different inhibitors is not identical. A PD-L1 assay is now routinely performed for cancer patients to determine whether they are eligible for pembrolizumab treatment. The KEYNOTE-024 study, together with KEYNOTE-042 study, demonstrated that a PD-L1 tumor proportion score (TPS) ≥50% should act as the cut-off value to select patients with advanced NSCLC to receive first-line pembrolizumab monotherapy, while the standard second-line pembrolizumab monotherapy requires patients to have a TPS ≥1% (57, 62). Notably, the predictive role of PD-L1 expression for nivolumab is not as effective as that for pembrolizumab. In the Checkmate-032 study, patients with recurrent or metastatic urothelial cancer who were treated with nivolumab showed no significant difference in objective response rate (ORR) between the subgroup with PD-L1 expression levels ≥1% and that <1% (24.0 vs. 26.2%, respectively) (63). Likewise, compared with chemotherapy, the CheckMate 026 study also showed no improvement in PFS or overall survival (OS) of patients with untreated stage IV or recurrent NSCLC and a PD-L1 expression level ≥5% who treated with nivolumab (mPFS 4.2 vs. 5.9 mons, *p* = 0.25; mOS 14.4 vs. 13.2 mons) (64). Of particular interest, subsequent exploratory analysis of this trial has shown that TMB is a good predictor for nivolumab treatment efficacy. It demonstrated that patients with a high TMB had superior mPFS and ORR with nivolumab vs. chemotherapy (9.7 vs. 5.8 months, 47 vs. 28%, respectively).

One explanation for the difference in the predictive power of PD-L1 expression between different inhibitors may be attributed to the assessment of PD-L1 expression. So far, the immunohistochemistry (IHC) assay for each inhibitors varies (65). Hence, chances are that for the same sample, the assessment of PD-L1 status may be inconsistent when conducted by different assay. Whether different IHC assays share equivalent measurement performance and interchangeably remains to be deeply investigated. In order to better select the population that would receive optimal benefit, further studies are required to determine whether PD-L1 expression and TMB are two independent biomarkers with different predictive values for different drugs. If so, what are the specific mechanisms that cause this difference? Moreover, as more and more predictive biomarkers such as mismatch-repair deficiency and tumor infiltrating immune cells, are being studied, whether they have different predictive power for different inhibitors should be taken more consideration as well.

### Clinical Efficacy

Currently, PD-1/PD-L1 inhibitors have been approved for the treatment of various tumors, including NSCLC, melanoma, urothelial cancer, esophageal cancer, RCC, and Hodgkin's lymphoma (55, 56, 66–75). Although all PD-1/PD-L1 inhibitors work by targeting the same PD-1/PD-L1 signaling pathway, whether the clinical efficacy is comparable between different inhibitors remains unclear. In a preclinical trial, Tyler J. Curiel et al. made a direct comparison of PD-1 inhibitors and PD-L1 inhibitors to explore their efficacy in aged host treatment. The contrast revealed that PD-1 inhibitors could treat B16 tumors in aged hosts as effectively as in young hosts whereas PD-L1 inhibitors failed to exert potent anti-tumor effect in the same condition of aged hosts (76). Attention was greatly aroused whether difference of the efficacy between inhibitors exist in clinical practice.

Take the treatment of NSCLC for example, based on currently available clinical data, there are differences in clinical treatment options for each inhibitor, which are mainly attributed to the potential differences in clinical efficacy (Table 3) (55, 57, 62, 64, 66–68, 77–87). For first-line monotherapy for NSCLC, the results of the KEYNOTE-024 and KEYNOTE 042, showed that pembrolizumab has superior survival benefits, when compared with chemotherapy, in advanced NSCLC patients (57, 62). And in the latest interim OS analysis of the IMpower110, it was demonstrated that atezolizumab treatment resulted in longer PFS and OS than chemotherapy in the cohort with TC3 or IC3 of untreated metastatic NSCLC (77). However, in the phase 3 study, CheckMate 026, involving patients with previously untreated stage IV or recurrent NSCLC, nivolumab did not appreciably improve mOS when compared with chemotherapy (14.4 vs. 13.2 months, respectively) (64). Except for monotherapy, PD-1 or PD-L1 inhibitors combined with other anti-tumor treatments for first-line NSCLC therapy have also shown promising results and several combination treatments have been approved by the FDA for clinical practice. In the phase 3 KEYNOTE 189 and KEYNOTE 407 trials of patients with untreated metastatic NSCLC, pembrolizumab combined with chemotherapy resulted in significantly longer OS and PFS when compared with chemotherapy alone (78, 79). Instead of combining with chemotherapy, first-line nivolumab plus ipilimumab for advanced NSCLC treatment has been shown to have obvious survival benefits as well, with significantly longer PFS and improved ORR (7.2 vs. 5.5 mons, 45.3 vs. 26.9%, respectively) (80). However, the published IMpower131 and IMpower132 trial of atezolizumab showed no statistically significant difference in OS (82, 83). Of particular concern, a further comparison of the KEYNOTE407 and IMpower131 studies showed that pembrolizumab plus chemotherapy led to superior OS and PFS than atezolizumab plus chemotherapy, especially in PD-L1-low/negative patients. Notably, until now, nivolumab, pembrolizumab, and atezolizumab have all been widely used for second-line treatment of patients with disease recurrence or progression after prior platinum-based regimens and this treatment strategy has achieved impressive survival benefits (Table 3) (55, 56, 66–68). However, it has been observed that avelumab does not improve survival compared with docetaxel, in patients with prior platinum-treated PD-L1-positive NSCLC (87).

**Table 3:** The results of recent advances in landmark trials of different PD-1/PD-L1 inhibitors for the treatment of lung cancer.

**Drugs**	**Trials**	**NCT number**	**Patients**	**Trial design**	**Efficacy and safety**				**References**
					**ORR**	**PFS**	**OS**	**AE3-5**	
**First-line monotherapy**									
Pembrolizumab	KEYNOTE-024	NCT02142738	305	Pembrolizumab vs. platinum-based chemotherapy (PD-L1 >50%)	44.8 vs. 27.8%	mPFS 10.3 vs. 6.0 *p* < 0.001	estimated 6-mons OS 80.2 vs. 72.4% *p* = 0.005	26.6 vs. 53.3%	(57)
	KEYNOTE-042	NCT02220894	1,274	Pembrolizumab vs. platinum-based chemotherapy (PD-L1 >1%)	27 vs. 27%	mPFS 5.4 vs. 6.5	mOS 16.7 vs. 12.1 *p* = 0.0018	18 vs. 41%	(62)
Atezolizumab	IMpower 110	NCT02409342	572	Atezolizumab vs. platinum-based chemotherapy (TC3 or IC3)	38.3 vs. 28.6	6-mons PFS 59.8 vs. 39.3 12-mons PFS 36.9 vs. 21.6	mOS 20.2 vs. 13.1 *p* = 0.0106	31.8 vs. 53.6%	(77)
Nivolumab	CheckMate 026	NCT02041533	423	Nivolumab vs. platinum-based chemotherapy (PD-L1 ≥5%)	26 vs. 33%	mPFS 4.2 vs. 5.9 *p* = 0.25	mOS 14.4 vs. 13.2	18 vs. 51%	(64)
**First-line combination therapy**									
Pembrolizumab	KEYNOTE-189	NCT02578680	616	Pembrolizumab+chemotherapy vs. chemotherapy	47.6 vs. 18.9% *p* < 0.001	mPFS 8.8 vs. 4.9 *p* < 0.001	Estimated 1-year OS 69.2 vs. 49.4% *p* < 0.001	67.2 vs. 65.8%	(78)
	KEYNOTE-407	NCT02775435	559	Pembrolizumab+chemotherapy vs. chemotherapy	57.9 vs. 38.4%	mPFS 6.4 vs. 4.8 *p* < 0.001	mOS 15.9 vs. 11.3 *p* < 0.001	69.8% vs. 68.2%	(79)
Nivolumab	CheckMate 227	NCT02477826	1,739	Nivolumab+ipilimumab vs. chemotherapy	45.3 vs. 26.9%	mPFS 7.2 vs. 5.5 *p* < 0.001	NR	31.2 vs. 36.1%	(80)
Atezolizumab	IMpower 130	NCT02367781	723	Atezolizumab+chemotherapy vs. chemotherapy	49.2 vs. 31.9%	mPFS 7.0 vs. 5.5 *p* < 0.0001	mOS 18.6 vs. 13.9 *p* = 0.033	81 vs. 71%	(81)
	IMpower 131	NCT02367794	1,021	Atezolizumab+chemotherapy vs. chemotherapy	NR	mPFS 6.3 vs. 5.6	mOS 14.2 vs. 13.5	68 vs. 57%	(82)
	IMpower 132	NCT02657434	578	Atezolizumab+chemotherapy vs. chemotherapy	47 vs. 32%	mPFS 7.6 vs. 5.2	1-year-OS 59.6 vs. 55.4%	69 vs. 59%	(83)
	IMpower 150	NCT02366143	692	Atezolizumab+chemotherapy+bevacizumab vs. chemotherapy+bevacizumab	63.5 vs. 48.0%	mPFS 8.3 vs. 6.8 *p* < 0.001	mOS 19.2 vs. 14.7 *p* = 0.02	58.5 vs. 50.0%	(84)
**Second-line therapy**									
Pembrolizumab	KEYNOTE-010	NCT01905657	1,034	pembrolizumab 2 mg/kg vs. docetaxel; pembrolizumab 10 mg/kg vs. docetaxel	18 vs. 9%; 18 vs. 9% PD-L1 >50% 30 vs. 8%; 29 vs. 8%	mPFS 3.9 vs 4.0 *p* = 0.07; 4.0 vs. 4.0 *p* = 0.004 PD-L1 >50% 5.0 vs. 4.1 *p* = 0.0001; 5.2 vs. 4.1 *p* < 0.0001	mOS 10.4 vs. 8.5 *p* = 0.0008; 12.7 vs. 8.5 *p* < 0.0001 PD-L1 >50% 14.9 vs. 8.2 *p* = 0.0002; 17.3 vs. 8.2 *p* < 0.0001	13 vs. 16%; 13 vs. 35%	(55)
Nivolumab	CheckMate 017	NCT01642004	272	Nivolumab vs. docetaxel	20 vs. 9% *p* = 0.0083 = 0.0083	mPFS 3.5 vs. 2.8 *p* < 0.001	mOS 9.2 vs. 6.0 *p* < 0.001	7 vs. 57%	(66)
	CheckMate 057	NCT01673867	582	Nivolumab vs. docetaxel	19%vs. 12% *p* = 0.02	mPFS 2.3 vs. 4.2	mOS 12.2 vs. 9.4 *p* = 0.002	10 vs. 54%	(67)
	CheckMate 078	NCT02613507	504	Nivolumab vs. docetaxel	16.6 vs. 4.2% *p* < 0.0001	mPFS 2.8 vs. 2.8 *p* = 0.0147	mOS 12.0 vs. 9.6 *p* = 0.0006	10 vs. 48%	(85)
Atezolizumab	OAK	NCT02008227	1,225	Atezolizumab vs. docetaxel	14 vs. 13%	mPFS 2.8 vs. 4.0 *p* = 0.49	mOS 13.8 vs. 9.6 *p* = 0.0003	15 vs. 43%	(68)
	POPLAR	NCT01903993	287	Atezolizumab vs. docetaxel	57 vs. 24% in the TC3 or IC3 subgroup 38 vs. 13%	mPFS 2.7 vs. 3.0 in the TC3 or IC3 subgroup 7.8 vs. 3.9	12.6 vs. 9.7 *p* = 0.04 in the TC3 or IC3 subgroup 15.5 vs. 11.1	11 vs. 39%	(86)
Avelumab	JAVELIN Lung 200	NCT02395172	792	Avelumab vs. docetaxel (PD-L1-positive)	19 vs. 12% *p* = 0.011	mPFS 3.4 vs. 4.1 *p* = 0.53	mOS 11.4 vs. 10.3 *p* = 0.16	10 vs. 49%	(87)

Several pooled analyses have made indirect comparisons among PD-1/PD-L1 inhibitors. A comparative analysis by Pillai et al. showed that the ORR in the population treated with PD-1 inhibitors was not significantly different from that in the group treated with PD-L1 inhibitors (19 vs. 18.6%; *p* = 0.17) (88). However, a meta-analysis of five trials demonstrated that nivolumab and pembrolizumab exhibit a superior ORR compared to atezolizumab (89). Nonetheless, they are not remarkably different in either PFS or OS. A pooled statistical analysis of randomized, controlled trials performed by Carbognin et al. indirectly compared the efficacy of PD-1 inhibitors in the treatment of NSCLC, advanced melanoma and genitourinary cancer. The results showed that the ORRs of nivolumab-treated patients in PD-L1-positive and -negative subgroups were 39.3% (34.1–44.4%) and 22.9% (19.4–26.3%), respectively, while the corresponding rates in pembrolizumab-treated patients were 30.3% (25.2–35.3%) and 10.8% (3.3–18.4%), respectively (90). Moreover, in a pooled analysis of second- or later-line treatment of nasopharyngeal cancer, camrelizumab (34.1%) exhibited the highest ORR, followed by pembrolizumab (26.3%), JS001 (23.3%), and nivolumab (19.0%) (91). Besides, Duan J et al. conducted a meta-analysis with an adjusted mirror principal to explore the potential differences in PD-1/PD-L1 inhibitors. It showed that PD-1 inhibitors could achieve superior survival outcomes than PD-L1 inhibitors in patients with solid tumors irrespective of monotherapy, or combination treatment (92).

Although limited data obtained from large-scale clinical trials have provided some valuable information, no head-to-head clinical study has been performed to directly compare the efficacy of each PD-1/PD-L1 inhibitor. The inconsistencies in the reported clinical efficacy of PD-1/PD-L1 inhibitors warrants further investigation to determine whether these differences are drug-dependent or drug-independent, or are attributed to discrepancies in trial design or execution, or occur just by chance.

### Safety

In addition to escaping immune surveillance and developing malignancies, the PD-1/PD-L1 pathway can also mediate immunological homoeostasis (93). Therefore, while studying the anti-tumor benefits of PD-1/PD-L1 inhibitors, attention must be paid to the accompanying immune-related adverse effects (AEs). Immune-related AEs have been observed for nearly all of the PD-1/PD-L1 inhibitors (Table 3). They include fatigue, decreased appetite, diarrhea, hypothyroidism, hyperthyroidism, pneumonitis, skin reactions, and colitis (91, 94). In addition to excessive immune activation, another important cause of antibody-induced AEs is off-target binding. The antibodies are likely to cross-bind to functionally distinct, but structurally similar targets, leading to unexpected responses. However, when compared with traditional treatments, such as chemotherapy, PD-1/PD-L1 inhibitors have a significantly lower incidence of AEs. Typically, immune-related AEs are well-controlled by symptomatic treatment. Nonetheless, severe, fatal AEs can occur (95).

Nivolumab has been shown to have a narrow and mild toxicity spectrum, mainly causing endocrine toxicities (96). Atezolizumab is associated with a relatively high risk of developing hypothyroidism, nausea, and vomiting. Arthralgia, pneumonitis, and hepatic toxicities are the most common AEs caused by pembrolizumab (96), whereas for sintilimab, the most frequently reported AEs are hypothyroidism, increased blood thyroid-stimulating hormone levels, and decreased free thyroxine levels (3). Of particular concern, the PD-1 inhibitor, camrelizumab (SHR-1210), has been observed to have a highly specific AE known as capillary hemangioma, which appears after the initiation of treatment and regresses spontaneously both during and after treatment, with no remarkable effects on the anti-tumor efficacy or safety of the drug (97).

A pooled analysis performed by Xu et al. showed that the incidence of grade 1–5 AEs is 66.4, 71.8, and 75.1% for atezolizumab, nivolumab, and pembrolizumab, respectively, while the corresponding grade 3 or 4 AE incidence is 15.1, 14.1, and 19.8%, respectively (96). Moreover, a pooled analysis of nasopharyngeal cancer patients showed that the incidence rates of grade 1–5/3–5 AEs are 74.1/29.6, 54.2/17.4, 92.3/24.5, and 96.8/16.1% for pembrolizumab, nivolumab, JS001, and camrelizumab, respectively. This further indicates that, with respect to grade 1–5 AEs, nivolumab and pembrolizumab may be superior options, but when considering grade 3–5 AEs, camrelizumab and nivolumab show more favorable safety (91).

Due to limited data, population-based studies or large-scale head-to-head comparative trials are warranted to determine whether there are differences in the incidence and severity of AEs among different PD-1/PD-L1 inhibitors.

## Conclusion

As an increasing number of PD-1/PD-L1 inhibitors are used for the clinical treatment of cancer, there is a growing concern about whether they are comparable. It is well-known that both PD-1 inhibitors and PD-L1 inhibitors share the same rationale, in that they work by targeting and blocking the PD-1/PD-L1 signaling pathway. Nonetheless, these inhibitors are not completely interchangeable in clinical applications.

Based on the specific requirements of their effector functions, PD-1 inhibitors and PD-L1 inhibitors have a different structural basis, namely IgG4 and IgG1, respectively. Even with the same structural basis, it has been shown that characteristics such as the binding site, binding area, and binding affinity vary among the inhibitors. Although influenced by various factors, especially the degree of humanization, whether there is a significant difference in immunogenicity among inhibitors remains to be further studied. Moreover, the comparison of the clinical applications of these inhibitors, including predictive biomarkers, clinical efficacy, and safety provides insights for the design of future clinical research. In the era of precision medicine, in order to better select the population that will achieve optimal benefit, further studies are required to determine whether PD-L1 expression and TMB are two independent biomarkers with different predictive value for different drugs. Currently, clinical trials have shown significant differences in the selection of the therapeutic application and the corresponding efficacy of various inhibitors. Pooled analyses also revealed possible differences in clinical efficacy among the inhibitors. However, these indirect, non-head-to-head comparisons have certain limitations. It has been demonstrated that most inhibitors are well-tolerated, with nearly similar toxicity spectra. Nonetheless, the incidence and severity of various AEs associated with each inhibitor may vary to some extent. Whether this difference has remarkable clinical significance requires further investigation.

In summary, with the limited data available, this review compares the currently approved PD-1/PD-L1 inhibitors and provides some direction for future clinical studies. Population-based studies or large-scale head-to-head comparative trials are warranted to gain a more in-depth understanding of whether there are significant differences among PD-1/PD-L1 inhibitors and whether these potential differences directly affect their efficacy and safety, thus influencing the choice of the optimal population for clinical application.

## Author Contributions

XM designed the study, edited and approved final manuscript. JY made critical appraisal and approved final manuscript. JL, YP, and ZH collected the materials. YC analyzed the materials and drafted the article. All authors read and approved the final manuscript and contributed in the preparation of this work.

## Conflict of Interest

The authors declare that the research was conducted in the absence of any commercial or financial relationships that could be construed as a potential conflict of interest.

## Abbreviations

PD-1: Programmed cell death-1
PD-L1: Programmed death-ligand 1
FDA: Food and Drug Administration
NSCLC: Non-small cell lung cancer
IFN-γ: interferon-γ
ADCP: Antibody-dependent cellular phagocytosis
ADCC: Antibody-dependent cell-mediated cytotoxicity
CDC: Complement-dependent cytotoxicity
FAE: Fab-arm exchange
S228P: Serine228Proline
ADA: Anti-drug antibodies
PK: Pharmacokinetics
FcRn: neonatal Fc receptor
FcγR: Fcγ receptor
TMDD: Target-mediated drug disposition
Ag: Antigen
Ab: Antibody
NK cell: Natural killer cell
MAC: membrane attack complex
TMB: Tumor-mutation burden
PFS: Progression-free survival
TPS: Tumor proportion score
ORR: Objective response rate
OS: Overall survival
mPFS: median PFS
mOS: median OS
IHC: Immunohistochemistry
RCC: Renal cell carcinoma
AEs: Adverse effects.
